# Complete genome sequence of *Fervidobacterium gondwanense* Kr-7 isolated from a hot spring in Japan

**DOI:** 10.1128/mra.00899-25

**Published:** 2025-12-16

**Authors:** Tamotsu Kanai, Yuzuki Uonomi, Daichi Tanaka, Maito Hosonuma, Takeru Aiba, Shunya Kariya, Kouta Fujishima, Takara Doteuchi, Hiraku Takada, Ryota Sugimoto, Ryotaro Oiko, Shuto Uchiyama, Takashi Abe, Taku Oshima

**Affiliations:** 1 Graduate School of Engineering, Toyama Prefectural University, Kurokawa 57948, Imizu City, Toyama, Japan; 2 Department of Electrical and Information Engineering, Graduate School of Science and Technology, Niigata University 12978, Niigata City, Niigata, Japan; DOE Joint Genome Institute, Berkeley, California, USA

**Keywords:** *Fervidobacterium gondwanense*, Gram-negative bacteria, thermophilic bacteria, fermentation, hydrogen production

## Abstract

*Fervidobacterium gondwanense* Kr-7 strain (NBRC 117357) was isolated from a hot spring in Toyama Prefecture, Japan. This strain ferments a variety of organic compounds and produces CO_2_ and H_2_ as end products. Its genome sequence contains several hydrogenase genes that appear to be related to H_2_ production.

## ANNOUNCEMENT

*Fervidobacterium* is a genus of Gram-negative, anaerobic, thermophilic bacteria belonging to the phylum *Thermotogota* (1). They are heterotrophs that ferment various organic compounds, such as carbohydrates or protein-rich substrates, producing CO_2_ and H_2_ as end products.

Surface water samples from the Kuronagi hot spring in Toyama Prefecture, Japan, which were kept 1 week anaerobically, were used for anaerobic cultivation at 65°C in a YT medium (0.5% [w/v] yeast extract, 0.5% [w/v] tryptone, and 0.1% [w/v] NaCl). A bacterium from a single colony on a 1.5% agar-containing YT plate was rod-shaped, obligate anaerobic, and grew optimally at 65°C (detailed information on the isolation appears in https://www.ncbi.nlm.nih.gov/biosample/SAMD00916350). The strain produces CO_2_ and H_2_ determined by gas chromatography. Here, we present the whole genome sequence of the *F. gondwanense* Kr-7 (hereafter, abbreviated Kr-7).

Genomic DNA was obtained using Genomic-tips (Qiagen) from cells cultivated anaerobically in YT medium at 65°C for 24 h and sequenced at the Bioengineering Lab. (Japan). The genomic DNA was digested with g-TUBE (Covaris) to 10–20 kbp. The SMRTbell Express Template Prep Kit 2.0 and the Sequel IIe (PacBio) were used for library construction and sequencing. A total of 32,093 HiFi reads were generated using SMRT Link (PacBio, v11.0.0.146107; PacBio). Reads shorter than 1,000 bases were removed using Filtlong (v0.2.0) (2), and the remaining 27,010 reads were assembled using Flye (v2.9) (3). Then, the complete genome sequence was obtained. A genome quality assessment was conducted with CheckM (v1.2.3) (4) indicated a completeness of 99.20% and a 1.97% contamination level. The genome information is summarized in Table 1. The position of the first base pair of the genome was set at 100 bp upstream of the *dnaA* gene. To maintain accuracy, functional annotation was performed using DDBJ Fast Annotation and Submission Tool (5), employing two different programs: MetaGeneAnnotator (6) and Prodigal (v2.6.3) (7). Ribosomal RNA genes were predicted using Barrnap (v0.8) (8), and transfer RNA genes were predicted using tRNAscan-SE (v2.0.6) (9). The genome comprised 2,002 protein-coding genes, 6 rRNAs, 48 tRNAs, and 4 CRISPRs. All analyses were conducted using the default parameters.

**TABLE 1:** Genome information on *F. gondwanense* Kr-7.

Genome feature	Value for *F. gondwanense* Kr-7
Total sequence length (bp)	2,181,374
N50 (bp)	2,181,374
Genome coverage (X)	114.3
GC content (%)	39.7
Number of CDSs	2,002
Average protein length (aa)	333.2
Coding ratio (%)	91.7
Number of rRNAs	6
Number of tRNAs	48
Number of CRISPRs	4
Accession number	AP041034

**Fig 1:**
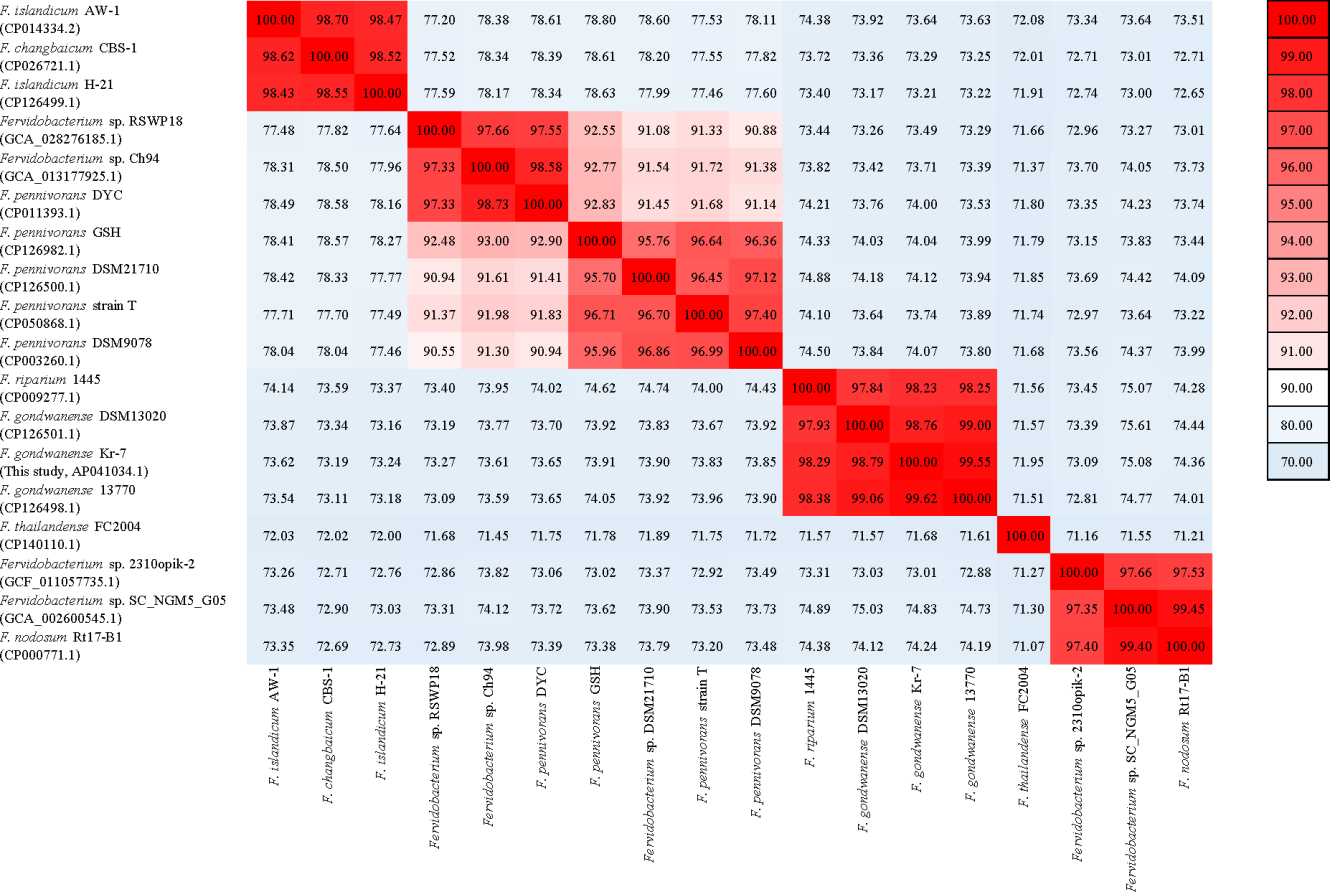
Heatmap of average nucleotide identity (ANI) values for 18 *Fervidobacterium* genomes, including Kr-7. The color bar is displayed on the right-hand side of the heatmap.

To confirm the taxonomic classification, a 16S rRNA-based phylogenetic analysis was conducted using the SINA (v1.2.12) web service (10), which revealed 94.73% sequence identity with members of the genus *Fervidobacterium*. Average nucleotide identity (ANI) analysis was performed using the *ANIb* option in PyANI (v0.2.10) (11) to compare the Kr-7 genome against 17 complete and draft genomes of *Fervidobacterium* (Fig. 1). The ANI values of 99.62% and 98.79% were observed between the Kr-7 and *F. gondwanense* 13770 and DSM 13020, respectively (Fig. 1).

As a heterotroph assimilating starch or carboxymethyl cellulose, the Kr-7 genome contains genes for glycolysis along with several amylases (e.g., TPNKR7_14240 and TPNKR7_18800) and an endoglucanase (TPNKR7_18210). [Fe-Fe] hydrogenase genes for H_2_ evolution (e.g., TPNKR7_03770) were identified along with a set of maturation proteins for constructing an active center (H-cluster).

Horizontal gene transfer has frequently been reported in hyperthermophilic bacteria of the genus *Fervidobacterium* (1, 12). Kr-7 contains 21 predicted transposase genes, 19 of which belong to the IS110 family.

## Data Availability

The whole genome sequence of strain Kr-7 has been deposited in DDBJ under accession number AP041034 (https://getentry.ddbj.nig.ac.jp/getentry/na/AP041034/). The raw sequencing data are publicly available in the Sequence Read Archive database under Run accession number DRR724714 and BioProject accession number PRJDB20789.
